# Protective Effect of *Raphanus sativus* Seed Extract on Damage Induced by In Vitro Incubation and Cryopreservation of Human Spermatozoa

**DOI:** 10.3390/antiox15010074

**Published:** 2026-01-06

**Authors:** Oumaima Ammar, Costanza Calamai, Mariachiara Marino, Elisabetta Baldi, Mario Maggi, Linda Vignozzi, Meriem Mehdi, Nadia Mulinacci, Monica Muratori

**Affiliations:** 1 Division of Obstetrics and Gynecology, Department of Health Sciences, University of Florence, Viale Pieraccini, 6, 50139 Florence, Italy; oumaima.ammar@unifi.it; 2 Department of Experimental and Clinical Biomedical Sciences “Mario Serio”, Viale Morgagni, 50, 50134 Florence, Italy; costanza.calamai@unifi.it (C.C.); mariachiaramarino12@gmail.com (M.M.); mario.maggi@unifi.it (M.M.); linda.vignozzi@unifi.it (L.V.); 3 Department of Experimental and Clinical Medicine, University of Florence, 50139 Florence, Italy; elisabetta.baldi@unifi.it; 4 Andrology, Women’s Endocrinology and Gender Incongruence Unit, AOU Careggi, 50139 Florence, Italy; 5 Centro di Ricerca e Innovazione sulle Patologie Surrenaliche, AOU Careggi, 50139 Florence, Italy; 6 Department of Histology, Embryology and Cytogenetics, University of Monastir, Monastir 5000, Tunisia; hbsmahdi@yahoo.fr; 7 Department of Neuroscience, Psychology, Drug and Child Health, Pharmaceutical and Nutraceutical Section, University of Florence, 50019 Florence, Italy

**Keywords:** sinapine, sinapoyl glycosides, *Raphanus sativus*, sperm in vitro incubation, sperm cryopreservation, sperm DNA fragmentation, oxidative stress, caspase activity

## Abstract

In vitro manipulation of human spermatozoa during Assisted Reproductive Technology (ART) can induce several damages to sperm structure and functions. This study investigated the protective effects of *Raphanus sativus* seed extract and its active compounds on several sperm parameters during in vitro incubation and cryopreservation. Extracts from five seed-batches were characterized by HPLC-DAD-MS and ^1^H-NMR, identifying sinapine and sinipic glycosides as the main characteristic compounds. Sperm DNA fragmentation (sDF) was detected by the Sperm Chromatin Dispersion test and LiveTUNEL. Excessive reactive oxygen species (ROS) production was detected by MitoSOX Red in viable spermatozoa. Caspase activity was detected by FLICA. Cryopreservation was conducted with two alternative freezing media. In vitro incubation with the extract protected against the loss of motility and reduced the induction of sDF, sperm ROS production, and caspase activity. Similarly, during cryopreservation, it allowed much better recoveries of sperm viability, motility, and DNA integrity by decreasing sperm ROS production with both freezing media. Sinapine and sinapic acid completely mimicked the protective effects of the whole extract during both in vitro incubation and cryopreservation, suggesting that they are included among the active principles. These findings support *Raphanus sativus* seed extract and its active compounds as candidates for inclusion in handling and freezing media for human spermatozoa in ART.

## 1. Introduction

In vitro manipulation of spermatozoa involves fundamental procedures for assisted reproductive technologies (ART), including in vitro incubation and cryopreservation. During In Vitro Fertilization (IVF), spermatozoa are incubated with the oocyte even for a long time, whereas cryopreservation is largely used to preserve fertility in cancer patients, other than for storing sperm from donors and, in certain patients, for ensuring that spermatozoa are available on the day of oocyte pick-up [1,2]. However, both cryopreservation and prolonged in vitro incubation are known to induce various sperm damages, including impaired motility and viability, lipid peroxidation, mitochondrial dysfunction, and DNA fragmentation [3,4]. Such alterations are commonly attributed to oxidative stress together with changes in membrane integrity and activation of apoptotic or regulated cell-death pathways [5,6,7,8,9]. In particular, during in vitro incubation, reactive oxygen species (ROS) generation may increase because sperm lose the antioxidant protection of seminal plasma and are subjected to laboratory handling conditions [8]. Similarly, in the context of cryopreservation, the freezing–thawing cycle subjects spermatozoa to osmotic and thermal stresses, thereby increasing ROS generation and triggering cryoinjury-associated pathways [5,6,7,9]. The biological and clinical consequences of damages induced by in vitro manipulation are significant, as they compromise sperm function and potentially affect fertilization and embryo development [10,11]. Hence, nowadays, there is an urgent need for safe, effective, and accessible strategies capable of minimizing sperm injury during ART procedures. In recent years, the potential of natural plant-derived compounds in protecting spermatozoa from iatrogenic damage was highlighted [12,13,14]. These molecules often exert multitarget effects, not only reducing oxidative stress but also stabilizing membranes and modulating cell survival pathways [15,16,17,18,19,20]. In particular, phenolic compounds have shown strong radical-scavenging capacity and low toxicity, making them promising candidates for inclusion in sperm preparation and preservation media [21,22]. Among these plants, *Raphanus sativus* L. (Brassicaceae), commonly known as radish, has long been used for both nutritional and therapeutic purposes. The leaves and root extracts of radish displayed anti-inflammatory, antimicrobial, anticancer, antimutagenic, and antioxidant effects [23,24,25,26], as well as protective actions against viral infections [27,28]. The review of Sham et al. (2013) [29] summarized the phytochemical composition and the pharmacological activities of *Raphanus sativus* seeds, highlighting that the ethanol extract exhibited marked antioxidant, antimicrobial, anti-inflammatory, and hepatoprotective properties, mainly attributed to the glucosinolate and phenolic constituents. Overall, research on the composition of *Raphanus sativus* seeds confirmed that the main phytochemicals are the alkaloid sinapine and the group of sinapic acid glucosides [29,30,31,32].

Despite the numerous reported bioactivities of this plant, in vitro data on the effects of *Raphanus sativus* seed extracts on human sperm functions relevant to ART remain limited. This study investigated the biological effects of an aqueous *Raphanus sativus* seed extract on human spermatozoa. Specifically, we aimed to: (i) chemically characterize the extract and verify the reproducibility of its profile across different seed batches; (ii) evaluate its ability to protect spermatozoa from damage induced by in vitro incubation; (iii) assess its protective role against cryodamage during freezing/thawing using two clinically relevant cryopreservation protocols. Furthermore, the biological effects of the whole extract were compared with those of two representative pure compounds, such as sinapine and sinapic acid.

## 2. Materials and Methods

### 2.1. Plant Material and Chemicals

Five seed samples of *Raphanus sativus* were purchased from local markets in Tunisia and in Tuscany. Two standards, sinapine chloride (purity grade by HPLC 99%), from hereon indicated as sinapine, and trans-sinapic acid (purity grade by HPLC 100%), from hereon indicated as sinapic acid, were purchased from Phytolab (GmbH & Co.KG, Dutendorfer, 91487 Vestenbergsgreuth, Germany). All the solvents used were of analytical purity grade; D_2_O was purchased from Sigma-Aldrich (Darmstadt, Germany).

### 2.2. Seed Extract Preparation

The seeds were ground with a coffee grinder (MoulinexCoffee Grinder-Two Stainless Steel Blades, 180W, 50G, MC300161, Moulinex, Groupe SEB, Écully, France), and the aqueous extracts were produced using the powdered seeds. For each batch, 5 g of dried material was extracted with 200 mL of water at 25 °C/overnight under stirring. After filtering, the solution was frozen and lyophilized by a freeze dryer Lyovac GT 2 (Leybold-Heraeus, Köln, Germany). The dried extract (DE) yield was 54.3 ± 4.3 mg/g of dried seeds. The dried sample was used for HPLC-DAD analyses and NMR experiments.

### 2.3. HPLC-DAD-MS Analyses

The dried extracts of the five samples were dissolved in ethanol/acidic water 7:3 (4–6 mg/mL, exactly weighted). The instrumentation electrospray (all from Agilent Corporation, Santa Clara, CA, USA) was a 1260 Infinity II LC System with a Diode Array and Mass Spectrometry Detectors, and API as the interface (In-finityLab LC/MSD). A Poroshell column (EC-C1, 120 A, 150 mm × 3.0 mm id, 2.7 µm of Agilent Technologies); the flow rate was 0.4 mL min-1. The binary mobile phase was constituted by acetonitrile (A) and water at pH 3.2, with formic acid (B). The following multistep linear gradient was applied: A 5% at time 0, A 40% at 40 min with a 5 min plateau, A 70% at 50 min with a plateau of 10 min and finally A 100% at 65 min with a plateau of 3 min; finally, the column returned to A 5% in 2 min. The chromatograms were recorded at wavelengths set at 220, 280, 330, and 350 nm. The mass spectra were acquired in negative and positive ion mode, mass range acquisition 100–1000 Dalton, gas temperature 350 °C, nitrogen flow rate 12 L/min, nebulizer pressure 35 psi, capillary voltage 3500 V, and fragmentation energy between 100 V and 300 V.

For the quantitative data, sinapine were determined from HPLC-DAD at 330 nm using the calibration curve of sinapine (R^2^ 0.999, linearity range 0–4.08 µg), and sinapic derivatives were evaluated at 330 nm by the calibration line of sinapic acid (R^2^ 0.997, linearity range 0–3.99 µg). Hypothesizing that the biological effects were attributable to the sinapoyl group, the concentration of sinapic acid was calculated as the sum of all sinapoyl glycosides, while sinapine was evaluated as such. To correlate the extract with the seed used for its preparation, the data were first expressed on a seed weight basis and then converted to mg/g of dried aqueous extract, taking into account the weight of the freeze-dried extract (DE) obtained from each gram of dried seed. Finally, the amounts of sinapic acid and sinapine corresponding to 25 µg of DE, the dose used for the bioassays, were calculated by proportion.

### 2.4. ^1^H-NMR Analyses

The proton spectra were recorded for each dried extract using an Advance 400 MHz Bruker instrument (Bruker, Bremen, Germany). For each sample, the final concentration was approximately 5 mg/mL of DE (exactly weighted) in D_2_O.

### 2.5. Reagents for Cellular Assays

Human Tubal Fluid (HTF), Human serum Albumin (HSA) was purchased by Fujifilm, Irvine Scientific (Rome, Italy). The Halosperm kit was obtained from Halotech DNA (Madrid, Spain). MitoSOX™ Red, LIVE/DEAD™Fixable Far Red Dead Cell Stain (LD-FR), LIVE/DEAD™Fixable Green Dead Cell Stain (LD-G), and Vybrant FAM caspase-3 and -7 assay kit were purchased from Thermo Fisher Scientific (Waltham, MA, USA). All other reagents were obtained from Merck Life Science (Milan, Italy), unless otherwise indicated.

### 2.6. Samples Collection and Preparation

Semen samples were collected according to the criteria of the World Health Organization (2021) [33] from subjects undergoing routine semen analysis at the Semen Cryopreservation and Andrology Laboratory of Careggi Hospital. Routine semen analysis was performed by determining the number, concentration, progressive and total motility, and morphology of spermatozoa by light microscopy. In addition, semen volume was evaluated by weighing the sample, and semen pH was determined by using pH paper and comparing the obtained color with the calibration strip. The laboratory where we collected semen samples participates in external quality control programs: the United Kingdom National External Quality Assessment Service (NEQAS) and the External Quality Assessment of Tuscany. All subjects aged ≥18 years and who provided signed informed consent were consecutively enrolled in the study. Subjects were excluded if they did not maintain sexual abstinence for 2–7 days before sample collection or reported semen loss at the time of collection. Additional exclusion criteria included leukocytospermia, presence of bacteria in semen, recent drug treatment, and smoking habits. Semen samples with insufficient sperm count to perform the planned analyses were also excluded.

In experiments of in vitro incubation, we used swim-up selected sperm samples. For these experiments, only samples with ≥30% progressive motility were included. Indirect swim-up was performed by washing 1 mL of semen with 1 mL of HTF, centrifuging for 5 min at 500× *g*, overlaying the pellet with 1 mL of fresh HTF-HSA1%, and incubating at 37 °C for 45 min. Hence, the motile fraction was counted and assessed for motility. Supplementary Table S1 reports the median values for age, abstinence, and standard semen parameters of subjects recruited for experiments of in vitro incubation.

For experiments of cryopreservation and for the detection of sDF by LiveTUNEL (see below), we used native semen samples consecutively collected. Supplementary Table S2 reports the median values for age, abstinence, and standard semen parameters of subjects recruited for experiments of cryopreservation.

### 2.7. In Vitro Incubation and Cryopreservation

In preliminary experiments aimed at determining the optimal concentration of the extract, we incubated swim-up selected sperm for 2 h with the following extract concentrations: 1, 5, 10, 25, 50, and 100 μg/mL [22]. After incubation, progressive and total motility were determined, with 25 μg/mL being the most effective concentration in protecting sperm from motility loss due to incubation (Supplementary Figure S1). Hence, the following experiments were conducted with this extract concentration. We used two experimental groups: one for in vitro incubation and the other for cryopreservation.

In vitro incubation (n = 22) was performed in HTF at 1 × 10^6^ sperm/100 µL at 37 °C and 5% CO_2_, with or without treatment with the extract (25 μg/mL), sinapine (0.376 μg/mL), or sinapic acid (0.282 μg/mL). Before and after incubation at the indicated times, motility, excessive ROS production, caspase activity, and sperm DNA fragmentation (sDF) were assessed.

For cryopreservation (n = 32), we used a Vapour Fast Freezing procedure. Aliquots (250 µL) of native semen were mixed 1:1 (*v*/*v*) with freezing medium, either Test Yolk Buffer (TYB, Fujifilm, Irvine Scientific, Rome, Italy) or SpermFreeze^®^ (SF, FertiPro, Beernem, Belgium). Hereon, the procedure using TYB was indicated as the TYB procedure, whereas the procedure using SF was indicated as the SF procedure. Where indicated, the freezing media were supplemented with 25 µg/mL *Raphanus sativus* seed extract or pure sinapine (0.376 μg/mL) or sinapic acid (0.282 μg/mL). The freezing medium was added gradually with gentle mixing. After 15 min equilibration at 37 °C, the mixture was aspirated into 500 μL security straws, subsequently sealed, and placed horizontally in the vapor phase of liquid nitrogen (LN2) in a floating support at 5 cm above the surface of LN2 for 8 min (cooling rate of 15.6 °C/min). Then, the straws were plunged and stored in LN2 (−196 °C). Samples were thawed by immersion in a 37 °C water bath for 15 min. Before and after cryopreservation, motility, viability, excessive ROS production, and sDF were assessed.

All the microscopic evaluations were conducted in a blinded manner.

### 2.8. Evaluation of Sperm Motility and Viability

Sperm motility was evaluated by placing 10 μL of the sperm suspension on a slide heated to 37 °C, covering it with a coverslip, and observing it under a phase-contrast microscope. Motility was evaluated on at least 200 spermatozoa and expressed as a percentage of progressive and in situ motility and immotile sperm.

Sperm viability was evaluated only before and after cryopreservation using the eosin staining test. Equal volumes of sperm suspension and 1% eosin solution were mixed, and at least 200 spermatozoa per sample were examined by optical microscopy. Viable spermatozoa remained unstained, while non-viable spermatozoa stained dark pink. In our hands, this test shows very low intra-assay coefficients of variation CVs [34].

### 2.9. Evaluation of sDF

SDF was determined by the sperm chromatin dispersion (SCD) test, using the Halosperm kit (Halotech DNA SL, Madrid, Spain)**.** Briefly, sperm were mixed with melted agarose, placed on an agarose-coated slide, covered with a coverslip, and gelled. Slides were immersed in acid and then in lysis solution (both provided by the kit) and then dehydrated in ethanol baths. Finally, slides were stained with eosin and subsequently with thiazine (15 min each) and examined under 100× light microscopy. At least 200 spermatozoa were scored for the presence/absence of a halo around the nuclear core [35]. In our hands, this test shows very low intra- and inter-assay CVs [34].

In some experiments, sDF was evaluated in viable spermatozoa by using the LiveTUNEL assay (Roche Diagnostics, Mannheim, Germany), a flow-cytometric method that combines LD-FR staining [36] with TUNEL (terminal deoxynucleotidyl transferase (TdT)-mediated fluorescein-dUTP nick end labeling), an assay with very low intra-assay CVs [37]. Fresh semen samples (2–8 × 10^6^ spermatozoa) were washed twice with HTF medium and incubated for 24 h at 37 °C in a 5% CO_2_ atmosphere either in the absence or in the presence of the extract. Before and after incubation, samples were stained with LD-FR (1:10,000 in PBS, 500 μL) for 1 h at room temperature in the dark, and then sDF was detected by TUNEL, using the In Situ Cell Death Detection Kit (Roche Diagnostics, Mannheim, Germany), fluorescein. To this aim, samples were washed and then fixed with 4% paraformaldehyde in PBS (30 min, RT). Hence, samples were washed twice with PBS containing 1% of bovine serum albumin, permeabilized in 100 µL of 0.1% Triton X-100/0.1% sodium citrate for 4 min on ice, and incubated with the TUNEL reaction mixture containing TdT enzyme and FITC-dUTPs (for 1 h at 37 °C in the dark). Following two washes, spermatozoa were counterstained with DAPI (1 μg/mL, 15 min, RT in the dark) and analyzed by flow cytometry (FACSCanto II, BD Biosciences, San Jose, CA, USA), equipped with a violet laser (405 nm), a blue laser (488 nm), and a red laser (633 nm) for excitation. Fluorescence was collected using 450/40, 530/30, and 660/20 base peak (BP) filters to detect blue (DAPI), green (FITC), and far-red (LD-FR) emissions, respectively. For each experiment, a negative control (without TdT enzyme) was included. Data acquisition was carried out by recording 5000 LD-FR negative events (i.e., viable spermatozoa) within a flame-shaped region (FR) in the forward scatter/side scatter (FSC/SSC) dot plot, which excludes debris and non-sperm cells while containing spermatozoa and apoptotic bodies [38]. For data analysis, within FR, we drew a gate around viable sperm, and within such a gate, we set a threshold in the TUNEL/LD-FR dot plot of the negative control, including about 99% of viable sperm. Then we copied the threshold in the TUNEL/LD-FR dot plot of the corresponding test sample, where the percentage of viable DNA-fragmented sperm was determined as the fraction beyond the threshold. Data analysis was conducted with the FACSDiva software, version 9.0.1 (BDBiosciences, San Jose, CA, USA).

### 2.10. Evaluation of Excessive Sperm ROS Production

Excessive ROS production was measured using a double labeling with MitoSOX red and LD-G, with fluorescence detected by flow cytometry, as previously reported [39]. Briefly, 6 × 10^6^ spermatozoa were washed twice with HTF medium, and the pellet was then resuspended in 500 µL of PBS, where 5 µL of LD-G were added (final dilution: 1:10,000). Then samples were incubated for 1 h at room temperature in the dark. After incubation, samples were twice washed with PBS and split into two aliquots (100 μL each): (i) test sample where 2 μM of MitoSOX Red was added, and (ii) negative control without MitoSOX Red addition. Samples were incubated at room temperature for 15 min in the dark. Sperm were then washed twice with PBS, and the pellet was resuspended in PBS before flow cytometry analysis. Samples were acquired by a FACScan flow cytometer (FACScan, BDBiosciences, San Jose, CA, USA) equipped with a 15 mW argon laser (488 nm). After proper compensation of spillover of LD-G into the MitoSOX Red channel and vice versa, LD-G fluorescence was detected by an FL-1 detector (515–555 nm), while MitoSOX Red was detected by an FL-2 detector (563–607 nm). For each sample, 5000 LD-G-negative events (viable spermatozoa) were recorded within FR. For data analysis, within FR, we drew a gate around viable sperm, and within such a gate, we set a threshold in the MitoSOX Red/LD-G dot plot of the negative control, including about 99% of viable sperm. Then we copied the threshold in the MitoSOX Red/LD-G dot plot of the corresponding test sample, where the percentage of viable sperm with excessive ROS production of total viable sperm was determined. Data analysis was conducted by CellQuest Pro software, version 5.2.1 (BD Biosciences, San Jose, CA, USA).

### 2.11. Evaluation of Caspase Activity

For detection of active caspases 3 and 7, we used Vybrant FAM Caspase-3 and -7 Assay Kit, as previously reported [40]. Briefly, after twice washing with PBS, sperm samples (2 × 10^6^) were split into two aliquots (150 μL of cell suspension in PBS) for: (i) test sample, where 1 μL of FAM-DEVD-FMK reagent (FLICA 150X) was added; (ii) negative control, where FLICA was not added. Hence, the two aliquots were incubated for 1 h at 37 °C and then twice washed with the washing buffer (provided by the kit). After washing, samples were fixed (10% formaldehyde), washed, and then stained with propidium iodide (PI, 50 μg/mL for 10 min). At the end of the procedure, samples were acquired by a FACScan flow cytometer, recording 8000 events within FR in FSC/SSC dot plot, and after compensation of spillover between PI and FLICA channels. PI and FLICA fluorescence were detected using the FL-2 and FL-1 detectors, respectively. For data analysis, within FR, we draw a region around PI-positive events (i.e., spermatozoa), and within such a gate, we set a threshold in the FLICA/PI dot plot of the negative control, including about 99% of sperm. Then we copied the threshold in the FLICA/PI dot plot of the corresponding test sample, where the percentage of sperm with active caspase was determined. Data analysis was conducted by CellQuest Pro software.

### 2.12. Statistical Analysis

Statistical analysis was performed through SPSS software version 24.0 for Windows. The Kolmogorov-Smirnov test was used to evaluate whether the analyzed variables followed a normal distribution. Since most variables showed a non-normal distribution, in tables, data were expressed as median [interquartile range (IQR)]. In the graphs, the mean values were also reported. To compare multiple groups (for each variable: control value before and after incubation or cryopreservation and value for treated sample), we used Friedman two-way analysis of variance by ranks followed by post-hoc pairwise comparisons using the Wilcoxon signed-rank test with Bonferroni correction for *p*-value adjustment. To compare TYB and SF procedures, for each variable, we calculated the recovery rate (RR): post-thaw value/fresh sample value. The RRs were then compared using the Mann-Whitney U test. For all statistical analyses, a *p*-value < 0.05 was considered statistically significant.

## 3. Results

### 3.1. Chemical Characterization of the Raphanus sativus Seed Extract

**Figure 1:**
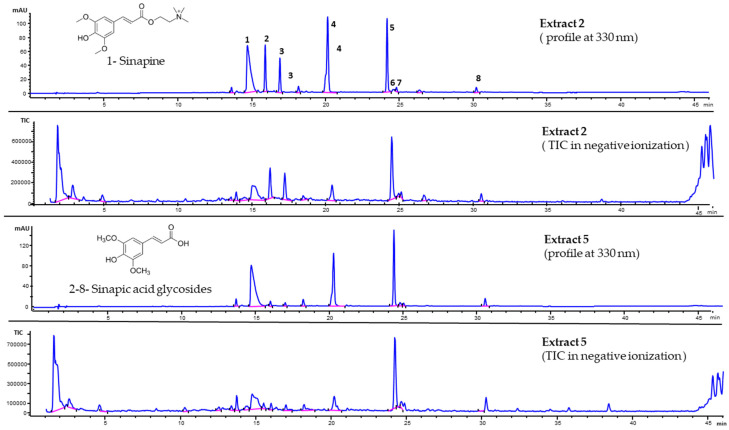
Chromatographic profiles of Extract 2 and Extract 5 at 330 nm and after negative ionization with fragmentor 180 V (TIC profile). The numbered compounds are according to Table 1.

To evaluate natural variability and ensure the repeatability of the extract’s chemical composition prior to the biological assays, five commercially available seed lots were compared. The aqueous extracts from seeds were chemically characterized by HPLC-DAD-MS and ^1^H-NMR spectroscopy. Chromatographic profiles at 330 nm of two of the five analyzed samples are compared in Figure 1.

Extract 2 and Extract 5 showed the same derivative, as also confirmed by the corresponding Total Ion Current (TIC) profiles in negative ionization mode. Overall, the UV and mass spectra, including their fragment ions in negative and positive ionization modes, along with the literature data [30,31,32], allowed the identification of the eight synapoyl derivatives listed in Table 1. The five seed batches consistently contained sinapine and several sinapic acid glycosides. Extracts 1, 2, and 3 exhibited a similar composition, whereas extracts 4 and 5 showed higher levels of the sinapic acid glycosides 5, 6, and 7 (Figure 1).

**Figure 2:**
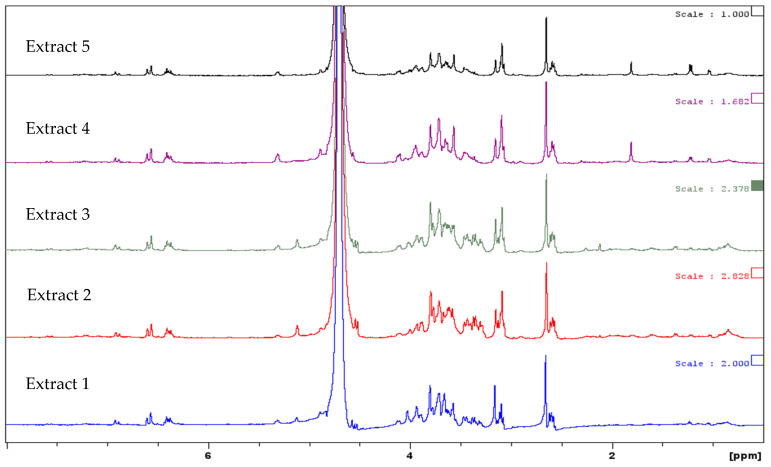
Comparison of the proton spectra in D_2_O of the aqueous extracts of the five seed batches of *Raphanus sativus*; the intense signal at 4.78 ppm is of H_2_O.

The ^1^H-NMR spectra, used to evaluate the presence of different organic molecules in the samples, showed signals in the same ppm ranges for all five extracts (Figure 2), confirming that sinapoyl derivatives were the predominant constituents of these dry extracts.

All the proton spectra showed the characteristic pattern of the aromatic protons at 6.0–7.5 ppm, confirming the presence of the sinapoyl group. Similarly, the presence of oligosaccharides of glycosidic derivatives was confirmed by the less intense signals at 5–5.5 ppm for the anomeric protons and at 3–4.3 ppm for the other sugar protons. Both HPLC and ^1^H-NMR analyses confirmed the similarity of the chemical profiles in the samples obtained from different seed batches. The same major compounds were identified in all samples, although in different relative amounts, as is typically observed when producing herbal extracts from different batches of raw material.

### 3.2. Quantitative Analysis of Phenolic Derivatives and Sinapine

**Figure 3:**
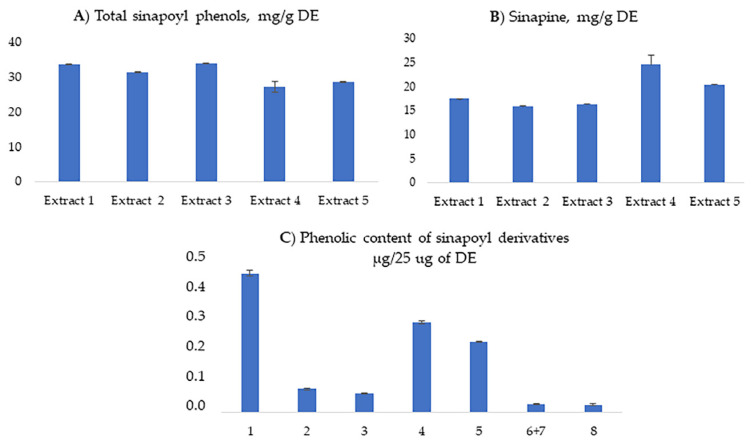
Quantitative determination by HPLC-DAD of: (**A**) Total sinapoyl glycosides; (**B**) sinapine content in the dry extracts obtained from five seed batches of *Raphanus sativus*; (**C**) content in Extract 1 of each sinapoyl derivative (1, sinapine and 2–8 sinapic acid glycosides—see Table 1) in the dose used for the bioassays. The data represent the means of triplicate.

The total synapoyl glycoside content, expressed as sinapic acid equivalents, ranged from 27.1 ± 1.6 mg to 33.9 ± 0.1 mg per g of DE (Figure 3A). The differences observed among the five extracts indicate low variability across seed batches.

The sinapine content in 1 g of DE ranged from 15.9 ± 0.1 mg to 24.6 ± 1.9 mg across batches, showing only a limited variability among seeds of different origin (Figure 3B).

Overall, the contents of sinapine and sinapic acid glycosides were consistent across all five batches analyzed, indicating a good reproducibility of the chemical profile for the aqueous extract.

Preliminary tests on human spermatozoa identified 25 μg/mL of DE as the optimal dose for the bioassays (see Section 2 for further details). Figure 3C summarizes the amount of sinapine and sinapic acid glycosides in this dose. To ensure consistency with the phytochemical composition of *Raphanus sativus* seed extract, the concentrations of the pure compounds used in the biological tests (sinapine and sinapic acid) were chosen to match those found in the extracts. The choice of sinapic acid as the pure compound to study is consistent with the hypothesis that the sinapoyl group represents the bioactive moiety of all sinapic acid glycosides identified in the extract. For testing the extract of biological parameters (see below), we used Extract 1.

### 3.3. Effects of the Extract During In Vitro Incubation

#### 3.3.1. Effect on Sperm Motility

**Figure 4:**
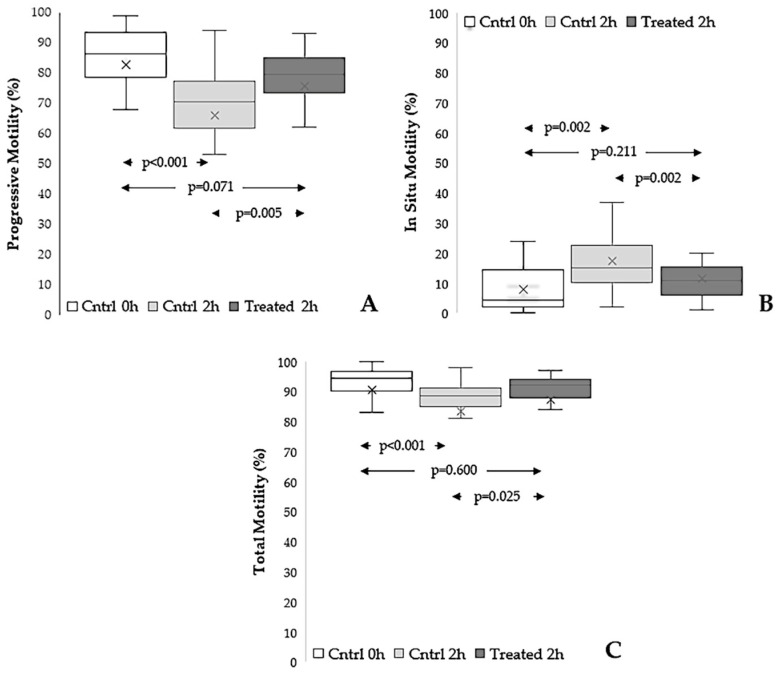
Effect of *Raphanus sativus* seed extract on sperm motility during 2 h of in vitro incubation. Boxplots reporting mean, median [IQR] and minimum and maximum values of progressive (**A**), in situ (**B**), and total motility (**C**) of swim-up–selected spermatozoa before (Cntrl 0 h), after 2 h incubation without extract (Cntrl 2 h), and after 2 h incubation with *Raphanus sativus* seed extract (Treated 2 h); n = 22.

As shown in Figure 4, incubation for 2 h of swim-up selected spermatozoa provoked a significant decrease in progressive motility (*p* < 0.001), and the presence of the extract significantly preserved such a parameter (*p* = 0.005), which, however, tended to remain lower than the baseline one (*p* = 0.071) (Figure 4A). The percentage of in situ motility was increased by 2 h of incubation (*p* = 0.002) and was significantly reduced by the presence of the extract (*p* = 0.002) (Figure 4B). Total motility was decreased by incubation (*p* < 0.001) and preserved in the sample treated by the extract (*p* = 0.025), where the parameter was not statistically different from the baseline value (*p* = 0.600) (Figure 4C).

#### 3.3.2. Effect on sDF

**Figure 5:**
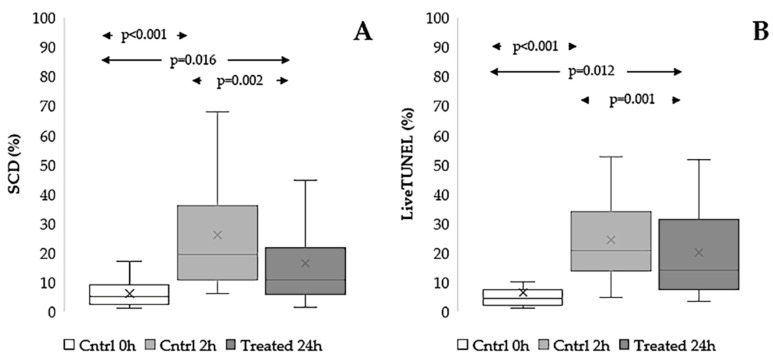
Effect of *Raphanus sativus* seed extract on sperm DNA fragmentation during 24 h of in vitro incubation. Boxplots reporting mean, median [IQR], and minimum and maximum values of sperm DNA fragmentation as detected by SCD test in swim-up selected spermatozoa, n = 22 (**A**) and by LiveTUNEL in the viable sperm fraction of native semen samples, n = 11 (**B**) before (Cntrl 0 h) and after 24 h incubation in untreated (Cntrl 24 h) and treated with the extract (Treated 24 h) samples.

The effect of the extract on sperm DNA integrity loss was tested after 24 h of in vitro incubation, a time sufficient to induce an increase of sDF, as shown in Figure 5 (*p* < 0.001 for both the SCD test and LiveTUNEL). In swim-up selected spermatozoa, the presence of the extract significantly reduced the amount of sperm DNA damage developed during incubation (*p* = 0.002), although it remained higher than the value before incubation (*p* = 0.016, Figure 5A). In some experiments, we tested the effect of the extract during in vitro incubation using native semen samples, focusing on the viable sperm fraction and detecting sDF by TUNEL (Supplementary Figure S2A, left panel). As shown in Figure 5B, these experiments confirmed the protective action of the extract on sperm DNA integrity.

#### 3.3.3. Effect on Excessive ROS Production

**Figure 6:**
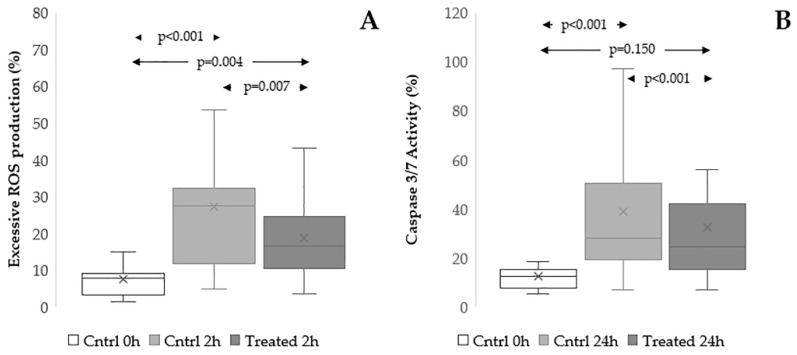
Effect of *Raphanus sativus* seed extract on excessive sperm ROS production and caspase activity during in vitro incubation. Boxplots reporting mean, median [IQR], and minimum and maximum values of excessive ROS production (**A**), n = 16, and caspase-3/7 activity (**B**), n = 19, before (Cntrl 0 h) and after incubation (2 h or 24 h) with (Treated 2 h or Treated 24 h) and without (Cntrl 2 h or Cntrl 24 h) *Raphanus sativus* seed extract.

To investigate the role of reduction of excessive ROS production in protective actions of the extract, we labelled swim-up selected spermatozoa by MitoSOX Red and LD-G before and after 2 h and 24 h of in vitro incubation (Supplementary Figure S2A, middle panel). Figure 6A shows that the amount of sperm ROS was highly increased after only 2 h of incubation (*p* < 0.001). The presence of the extract significantly reduced this parameter (*p* = 0.007), although the value remained higher than that before incubation (*p* = 0.004). After 24 h of in vitro incubation, all samples showed 100% of sperm with excessive ROS production, and the extract was ineffective.

#### 3.3.4. Effect on Sperm Caspase 3/7 Activity

To investigate the role of reduction of sperm apoptosis in protection of sperm DNA integrity, we evaluated sperm caspase 3/7 activity by FLICA/PI fluorescence analysis in swim-up selected spermatozoa before and after 24 h of in vitro incubation (Figure 6B and Supplementary Figure S2A, right panel). As shown, caspase activity highly increased during incubation (*p* < 0.001), and the extract produced a slight but significant reduction (*p* < 0.001), reaching a value not statistically different from that before incubation (*p* = 0.150).

#### 3.3.5. Effect of Sinapine and Sinapic Acid

As shown in Table 2, the addition of sinapine (0.376 µg/mL) or sinapic acid (0.282 µg/mL) thoroughly mimicked the actions of the extract. Indeed, both compounds protected spermatozoa from loss of progressive and total motility after 2 h and of DNA integrity after 24 h of incubation, with overlapping results to those of the whole extract. Excessive ROS production was reduced as well by both sinapine and sinapic acid, to a very close extent as the extract (Table 2).

### 3.4. Effects of the Extract on Cryodamage

The protective effects of the extract on cryodamage were tested in 32 subjects using a cryopreservation procedure with 500 μL straws as carriers and with TYB as the freezing medium. In a subgroup of these subjects (n = 17), the extract was tested using a similar procedure to cryopreserve sperm, but with SF as the freezing medium.

#### 3.4.1. Effect on Sperm Motility and Viability

**Figure 7:**
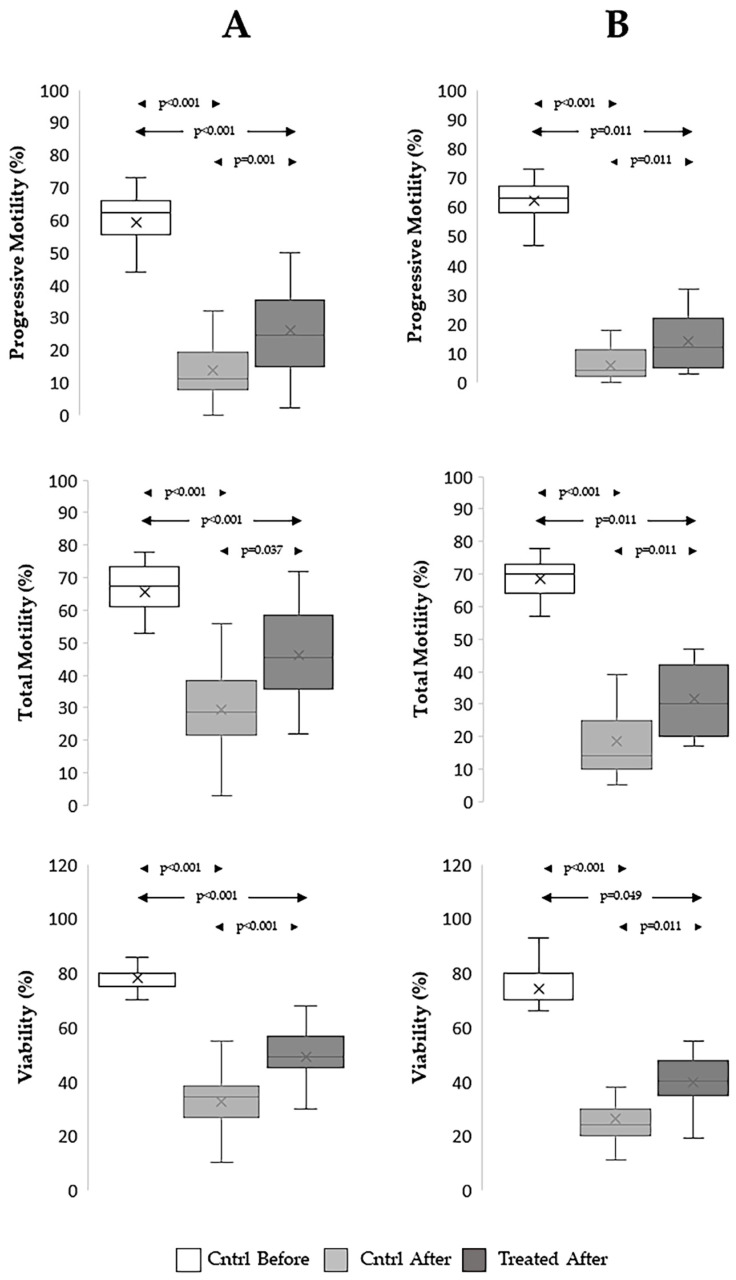
Effect of *Raphanus sativus* seed extract on sperm motility and viability during cryopreservation with TYB and SF procedures. Boxplots showing mean, median [IQR], and minimum and maximum values of progressive (**upper** panels) and total motility (**middle** panels) and viability (**lower** panels) of spermatozoa before (Cntrl Before) and after freezing–thawing without (Cntrl After) and with *Raphanus sativus* seed extract (Treated After). Samples were cryopreserved using TYB (**A**), n = 32, and SF procedures (**B**), n = 17.

As shown in Figure 7, freezing/thawing processes highly decreased progressive (*p* < 0.001 for both the TYB and SF procedures) and total motility (*p* < 0.001 for both the TYB and SF procedures) and viability (*p* < 0.001 for both the TYB and SF procedures) of spermatozoa. In our hands, the TYB procedure yielded better recoveries of the three parameters than the SF procedure, as shown by the comparison between corresponding RRs (Supplementary Table S3). However, irrespective of the procedure used, the addition of the extract significantly improved progressive (TYB procedure, *p* = 0.001; SF procedure, *p* = 0.011), total motility (TYB procedure, *p* = 0.037; SF procedure, *p* = 0.011), and viability (TYB procedure, *p* < 0.001; SF procedure, *p* = 0.011), although the values remained lower than those in fresh samples (Figure 7A: TYB procedure; Figure 7B: SF procedure).

#### 3.4.2. Effect on sDF

**Figure 8:**
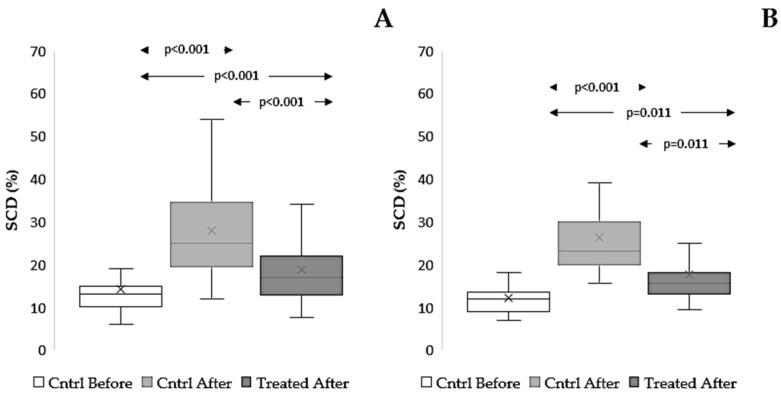
Effect of *Raphanus sativus* seed extract on sperm DNA fragmentation after cryopreservation. Boxplots showing mean, median [IQR], and minimum and maximum values of sperm DNA fragmentation as detected by the SCD test before (Cntrl Before) and after freezing–thawing without extract (Cntrl After) and with *Raphanus sativus* seed extract (Treated After). Samples were cryopreserved using TYB (**A**), n = 32, and SF procedures (**B**), n = 17.

As expected, cryopreservation procedures increased sDF levels in both cryopreservation procedures (TYB procedure, *p* < 0.001; SF procedure, *p* = 0.011) (Figure 8 and Supplementary Table S3). However, the extract was able to protect DNA from damage in both procedures (TYB procedure, *p* < 0.001: Figure 8A. SF procedure, *p* = 0.011: Figure 8B).

#### 3.4.3. Effect on Excessive ROS Production

**Figure 9:**
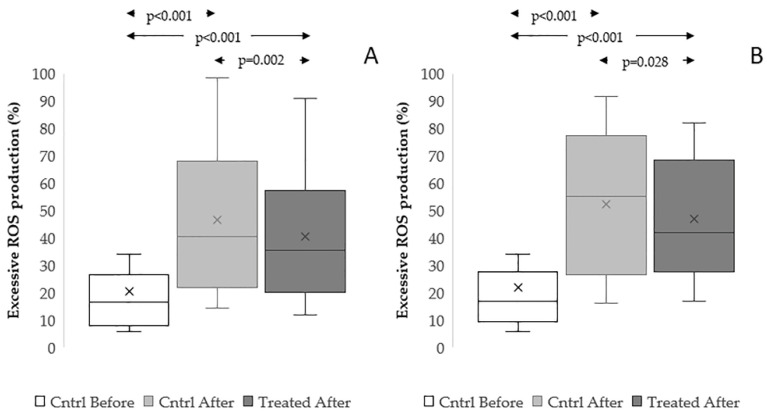
Effect of *Raphanus sativus* seed extract on excessive ROS production during cryopreservation. Boxplots showing mean, median [IQR], and minimum and maximum values of excessive ROS production before (Cntrl Before) and after freezing–thawing without extract (Cntrl After) and with *Raphanus sativus* seed extract (Treated After). Samples were cryopreserved using TYB (**A**), n = 32, and SF procedures (**B**), n = 17.

We studied the role of reduction of excessive ROS production in protective actions of the extract, by labelling viable spermatozoa with MitoSOX Red before and after cryopreservation (Figure 9 and Supplementary Figure S2B). We found that both procedures highly increased the percentage of sperm with excessive sperm ROS production (TYB procedure, *p* < 0.001: Figure 9A. SF procedure, *p* < 0.001: Figure 9B; Supplementary Table S3), whereas the addition of the extract provoked a decrease in the parameter with respect to the thawed control in both procedures (TYB procedure, *p* = 0.002. SF procedure, *p* = 0.028).

#### 3.4.4. Effect of Sinapine and Sinapic Acid

As shown in Table 3 and Table 4 (TYB and SF procedure, respectively), sinapine (0.376 µg/mL) and sinapic acid (0.282 µg/mL) resulted to in the protection of progressive and total motility, viability, and DNA integrity. In addition, such effects were overlapping with those obtained with the whole extract (Table 3 and Table 4).

## 4. Discussion

This study provides the first evidence that an aqueous seed extract of *Raphanus sativus* protects several functions of human spermatozoa during in vitro manipulation. Further, the study characterized the aqueous extracts for the first time by analyzing independent batches using HPLC and ^1^H-NMR. The results showed that the extracts were dominated by sinapine and several sinapic acid glycosides with good batch-to-batch homogeneity. The recurrent sinapoyl phytochemical signature indicated good reproducibility among batches.

Herbal preparations are intrinsically complex mixtures, containing multiple active or characteristic constituents, which may vary with several features, including the extraction method and the batch used. Hence, it is recommended that these preparations, when intended for experimental or clinical use, be chemically characterized in order to define their composition and reproducibility [41,42].

Chemical data confirmed the presence of sinapine as well as of eight glycosides of sinapic acid. Due to the use of water as an extraction solvent, all metabolites recovered from the seeds were highly soluble, and the samples showed no traces of lipophilic molecules, which are otherwise abundant in seeds. Quantitative analyses enabled the selection of two pure molecules, sinapine and sinapic acid, to partially represent the phytocomplex. Based on these data, it was possible to select doses of each standard mirroring the metabolite concentration present in 25 µg of DE, the extract amount identified as optimal for the bioassays. These compounds were used in parallel with the seed extract to confirm the hypothesized crucial role of the sinapoyl group in protecting human spermatozoa during in vitro manipulation (see below). Results obtained from chemical analyses are in agreement with previous phytochemical studies on *Rapanus sativus* seeds conducted on alcoholic or hydroalcoholic extracts, which consistently identify sinapine and sinapic-acid conjugates as dominant constituents, representing a defining phenolic motif of the seed matrix [29,30,31,32].

In biological tests, we found that the seed extract of *Raphanus sativus* showed protective actions towards sperm motility, viability, and DNA integrity during in vitro manipulation of spermatozoa. During in vitro incubation, the protective effect on DNA integrity was confirmed by two independent assays, SCD and LiveTUNEL, which together evaluate global chromatin abnormalities and DNA breaks in viable spermatozoa. LiveTUNEL, in particular, allowed us to test native semen samples but focusing on the viable sperm fraction and thus excluding non-viable spermatozoa that can fragment as well during incubation with non-specific processes. The observed protective actions were likely due to limiting the burst of oxidative stress provoked by incubation without the seminal antioxidant defenses [39,43,44], as suggested by the observed reduction in ROS production in viable sperm treated with the extract. Overall, our results are consistent with the established view that excessive ROS generated during sperm in vitro incubation can impair sperm quality and DNA integrity, and that adding exogenous antioxidants to sperm samples or media can buffer this redox stress and help preserve functional quality [18,45,46]. It has been reported that during in vitro incubation, oxidative stress also induces an apoptotic pathway leading to cell death [47]. Hence, we tested whether the extract was also able to reduce the activity of the effector caspases 3 and 7 by finding a small but significant decrease, consistent with the observed reduction in the sperm ROS production.

During in vitro incubation, other phytocomplexes have also been tested on human semen. Some extracts resulted in unsuccessful protection of human spermatozoa [48] or even showed spermicidal properties [49]. Conversely, extracts from *Tribulus terrestris* did protect motility, even if no effect was reported for DNA integrity [50]. Phytocompounds from *Capparis spinosa* have been shown to protect both motility and DNA integrity, but no effect on oxidative stress, as assessed by lipid peroxidation, was detected [21]. Overall, it appears that *Raphanus sativus* shows a broader spectrum of protective actions during in vitro incubation with respect to other tested plants.

When the seed extract of *Raphanus sativus* was added during cryopreservation, its protective action became even more evident. The damages observed in our experiments in thawed samples were consistent with the known mechanisms of cryodamage in human spermatozoa: rapid cooling and ice crystal formation induce osmotic and mechanical stress on the plasma membrane, mitochondrial activity is impaired, motility declines, and the freezing/thawing processes generate oxidative stress, which is closely associated with reduced vitality and accumulated sDF [6,7,51]. However, samples supplemented with the extract showed higher progressive and total motility, better viability, and lower levels of sperm ROS production and sDF, and these benefits were observed with both tested freezing media (TYB and SF). Comparable post-thawing improvements of motility, DNA integrity, and levels of oxidative stress have been described with other plant-derived supplements for human sperm cryopreservation, such as the extracts from *Olea europaea* [18], *Origanum vulgare* [52], *Terminalia arjuna* [53], and *Pinus massoniana* [54].

As mentioned, the chemical characterization of the extract allowed us to identify the presence of sinapine and several sinapic acid glycosides as the main characteristic components of the extract. Hence, we tested the corresponding standard molecules (i.e., sinapine and sinapic acid) individually at the concentrations of their presence in the extract. We found that the two standards completely mimicked the actions of the extract, both during in vitro incubation and cryopreservation, suggesting that the alkaloid sinapine and the residues of sinapic acid contributed to the protective properties of the seed extract of *Raphanus sativus.*

Overall, our study is consistent with the reports describing sinapic acid, sinapine, and related sinapoyl conjugates as antioxidant phenolic molecules in Brassicaceae seeds, displaying radical-scavenging and redox-buffering activity in vitro [55,56,57]. In the context of antioxidant strategies for in vitro sperm manipulation, earlier studies primarily evaluated single agents (i.e., vitamins, trace elements, coenzymes) and reported improvements in sperm quality and function [58,59,60,61]. More recently, attention has shifted toward whole phytocomplexes, in which multiple co-occurring constituents act synergically to protect sperm during in vitro manipulation, often yielding broader or stronger effects than single isolated compounds [12,18]. Seed extract of *Raphanus sativus* fits this rationale as a reproducible, aqueous, phenolic-rich extract suitable for supplementation during sperm preparation and freezing/thawing processes. With respect to previous studies on the effects of plant-derived extracts on human sperm [18,21,50,52,53,54], *Raphanus sativus* shows two features of particular translational interest. First, it is an aqueous extract with a defined composition and shows a demonstrated batch-to-batch reproducibility. Second, we report here that *Raphanus sativus* extract protects motility, viability, oxidative status, and DNA integrity both during incubation and after cryopreservation.

Plant-derived compounds/extracts have also been increasingly proposed as novel treatments for male infertility, a condition that affects about 7% of males and is expected to get worse in future years [62,63]. Some data present in the literature suggest that the seed extract of *Raphanus sativus* could also benefit male fertility in in vivo conditions. When the aqueous seed extract of *Raphanus sativus* was administered to mice with testis toxicity due to zearalenone, it counteracted the effects of the mycotoxin by improving sperm parameters and testosterone level, restoring testicular antioxidant enzymes, reducing lipid peroxidation, and limiting genotoxic alterations [64]. These findings suggest that compounds in the extracts are able to reach testicular sites at effective concentrations. Further, preparations from seeds and leaves of *Raphanus sativus* are used as herbal medicine for male infertility in Jordan [65], suggesting that the seed-derived compounds provide beneficial effects also in humans. In this regard, it is worth considering that *Raphanus sativus* seeds are included among the plant parts authorized for use in food supplements (e.g., the BELFRIT list adopted by France, Belgium, and Italy). Sinapic acid glycosides and sinapine are natural phenolic compounds widely found in Brassicaceae, plants that are extensively consumed in the human diet. To date, in vitro and in vivo studies have not reported any toxicity associated with sinapoyl glycosides or sinapine; conversely, several studies have highlighted their protective effects [55,66,67]. The extract shows a “food grade” profile, as only water was used for preparation, without any organic solvents or chemical reagents.

The study has some limitations. First, we used samples of overall good semen quality, and we cannot exclude that the protective effects of the extract and its compounds decrease/disappear in the case of very damaged samples. Secondly, although the plant and the seeds of *Raphanus sativus* are suitable for human consumption as described above, we didn’t perform a safety validation necessary for clinical use. Finally, in cryopreservation experiments, we tested the protective effects of the extract immediately after thawing without checking whether these effects extend over time in the thawed samples.

## 5. Conclusions

In summary, an aqueous extract of *Raphanus sativus* seed, chemically defined and reproducible across batches, preserved key functional parameters of human spermatozoa during in vitro incubation and cryopreservation. Sinapine and sinapic acid, determined as representative molecules, completely mimicked the protective actions of the whole extract. These findings support *Raphanus sativus* extract and its active compounds as a proper supplementation for sperm handling and freezing media in ART.

## 6. Patents

European Patent Register, n: WO2025062333; 2025.

## Figures and Tables

**Table 1:** List of the sinapoyl glycosides identified in the aqueous extract of the seeds of *Raphanus sativus*.

Compounds	RT min	Positive Ionization	Negative Ionization	mw
**1**—Sinapine	14.7	310, 251		310
**2**—Sinapoyl hexose	15.9	795 (2M^+^Na^+^), 409 (M^+^Na^+^), 369, 207	385, 325	386
**3**—Sinapoyl hexose	16.9	795 (2M^+^Na^+^), 409 (M^+^Na^+^), 369, 207	385, 325, 223	386
**4**—Sinapoyl derivative	20.1	725, 207, 175	715, 469, 223	-
**5**—Di-Sinapoyl-fructofuranosyl glucose	24.1	777 (M^+^Na^+^), 369, 207, 175	753, 547, 205	754
**6**—Di-Sinapoyl-fructo furanosyl glucose	24.5	859, 441, 287, 207	753, 417, 285	754
**7**—Sinapoyl-feruloyl fructo furanosyl glucose	24.8	747 (M^+^Na^+^), 287, 207	723, 417, 205	724
**8**—Tri-sinapoyl-fructo furanosyl glucose	30.3	983 (M^+^Na^+^), 575, 207	959	960

**Table 2:** Effect of the extract, sinapine, and sinapic acid on the indicated parameters during 2 or 24 h of in vitro incubation (n = 6). Cntrl 0 h, baseline control sample; Cntrl 2 h, control sample after incubation of 2 h; Cntrl 24 h, control sample after incubation of 24 h. Wilcoxon signed-rank test with Bonferroni correction for *p*-value adjustment. Data are median [IQR].

	Progressive Motility (%)	In Situ Motility (%)	Total Motility (%)	Excessive ROS Production (%)	sDF–SCD (%)
**Cntrl 0 h**	91.5 [75.25–96.75]	4.00 [1.00–16.25]	95.0 [90.00–100.00]	12.80 [8.67–26.88]	4.75 [2.13–8.25]
**Cntrl 2 h**	68.5 [54.00–76.50]	10.50 [7.50–20.75]	77.0 [74.75–84.00]	21.28 [12.93–53.44]	/
**Cntrl 24 h**	/	/	/	/	28.0 [22.25–35.75]
**Extract** *p* vs. *Cntrl 0 h* *p* vs. *Cntrl 2 h* *p* vs. *Cntrl 24 h*	77.5 [66.25–85.75] *0.225* *0.028* /	10.00 [5.00–21.75] *0.106* *0.891* /	88.5 [83.50–93.25] *0.081* *0.046* /	13.74 [10.36–39.05] *0.719* *0.043* /	18.75 [15.50–26.25] *0.285* / *0.027*
**Sinapine** *p* vs. *Cntrl 0 h* *p* vs. *Cntrl 2 h* *p* vs. *Cntrl 24 h* *p* vs. *Extract*	78.5 [63.75–87.25] *0.446* *0.027* / *1.000*	10.50 [5.25–22.50] *0.047* *0.916* / *1.000*	89.5 [84.75–93.25] *0.062* *0.046* / *1.000*	13.60 [11.10–35.79] *0.891* *0.043* / *1.000*	19.50 [15.13–25.75] *0.106* / *0.027* *1.000*
**Sinapic acid** *p* vs. *Cntrl 0 h* *p* vs. *Cntrl 2 h* *p* vs. *Cntrl 24 h* *p* vs. *Extract*	78.5 [62.75–85.25] *0.828* *0.028* / *1.000*	13.00 [5.50–25.25] *0.007* *0.223* / *1.000*	90.0 [85.75–94.25] *0.828* *0.046* / *1.000*	13.99 [11.03–35.89] *0.124* *0.043* / *1.000*	18.75 [13.38–24.00] *0.679* / *0.028* *1.000*

**Table 3:** Effect of the Extract, Sinapine, and Sinapic Acid on the indicated parameters during cryopreservation with TYB procedure (n = 5). Cntrl Before, control sample before cryopreservation; Cntrl After, control sample after cryopreservation. Wilcoxon signed-rank test with Bonferroni correction for *p*-value adjustment. Data are median [IQR].

	Progressive Motility (%)	Total Motility (%)	Viability (%)	sDF (%)
**Cntrl Before**	60.0 [56.0–64.5]	64.0 [63.5–69.0]	75.0 [70.0–77.5]	8.50 [7.50–11.75]
**Cntrl After**	15.0 [11.0–20.0]	31.0 [29.0–41.5]	37.0 [33.0–50.0]	19.00 [16.90–22.00]
**Extract** *p* vs. *Cntrl Before* *p* vs. *Cntrl After*	33.0 [19.0–38.5] *1.000* *0.043*	59.0 [45.5–63.0] *1.000* *0.043*	59.0 [52.5–62.0] *1.000* *0.043*	12.00 [10.50–16.00] *0.891* *0.042*
**Sinapine** *p* vs. *Cntrl Before* *p* vs. *Cntrl After* *p* vs. *Extract*	26.0 [16.5–37.5] *0.164* *0.043* *1.000*	56.0 [42.5–59.5] *0.455* *0.043* *1.000*	53.0 [50.0–60.0] *0.093* *0.043* *1.000*	13.00 [11.25–17.00] *0.574* *0.043* *1.000*
**Sinapic acid** *p* vs. *Cntrl Before* *p* vs. *Cntrl After* *p* vs. *Extract*	23.0 [19.5–37.5] *0.278* *0.042* *1.000*	52.0 [40.5–58.0] *0.069* *0.043* *1.000*	52.0 [48.0–62.5] *0.455* *0.043* *1.000*	13.00 [11.25–15.50] *0.164* *0.043* *1.000*

**Table 4:** Effect of the Extract, Sinapine, and Sinapic Acid on the indicated parameters during cryopreservation with SF procedure (n = 5). Cntrl Before, control sample before cryopreservation; Cntrl After, control sample after cryopreservation. Wilcoxon signed-rank test with Bonferroni correction for *p*-value adjustment. Data are median [IQ].

	Progressive Motility (%)	Total Motility (%)	Viability (%)	sDF (%)
**Cntrl Before**	60.0 [56.0–64.5]	64.0 [63.5–69.0]	75.0 [70.0–77.5]	8.50 [7.50–11.75]
**Cntrl After**	3.0 [2.0–11.5]	25.0 [15.5–34.5]	32.0 [22.5–40.5]	23.0 [16.0–23.5]
**Extract** *p* vs. *Cntrl before* *p* vs. *Cntrl after*	21.0 [6.0–25.0] *0.891* *0.043*	45.0 [25.0–46.5] *1.000* *0.043*	50.0 [33.5–55.0] *1.000* *0.043*	11.0 [10.0–14.5] *0.357* *0.042*
**Sinapine** *p* vs. *Cntrl before* *p* vs. *Cntrl after* *p* vs. *Extract*	12.0 [6.0–23.5] *0.357* *0.043* *1.000*	33.0 [23.0–45.0] *0.214* *0.068* *1.000*	42.0 [31.5–50.5] *0.124* *0.043* *1.000*	12.0 [10.0–13.5] *0.357* *0.041* *1.000*
**Sinapic acid** *p* vs. *Cntrl before* *p* vs. *Cntrl after* *p* vs. *Extract*	18.0 [4.0–24.0] *0.278* *0.042* *1.000*	32.0 [23.0–44.0] *0.051* *0.080* *1.000*	41.0 [32.0–51.0] *0.214* *0.043* *1.000*	11.0 [9.25–14.75] *0.719* *0.042* *1.000*

## Data Availability

The original contributions presented in this study are included in the article and Supplementary Materials. Further inquiries can be directed to the corresponding authors.

## Supplementary Materials

The following are available online at https://www.mdpi.com/article/10.3390/antiox15010074/s1, Table S1: Age, abstinence, and main semen parameters of subjects recruited for experiments of in vitro incubation (n = 33). Table S2: Age, abstinence, and main semen parameters of subjects recruited for cryopreservation experiments (n = 32). Table S3: Recovery rates of the indicated parameters by TYB and SF procedure for cryopreservation. Figure S1: Screening of Raphanus sativus seed extract concentrations (1–100 μg/mL). (A), progressive sperm motility: a, *p* (vs. C0h) < 0.001; b, *p* (vs. C2h) < 0.01. (B), total sperm motility: a, *p* (vs. C0h) < 0.05; b, *p* (vs. C2h) < 0.05. Data are mean ± SD; Wilcoxon signed-rank test with Bonferroni correction for *p*-value adjustment. C0h, Control before incubation; C2h, control after 2 h of incubation. Figure S2: (A), Treatment with the extract during in vitro incubation. Typical DAPI/TUNEL dot plots of the viable sperm fraction, obtained after gating of LD-FR negative events (left panel). Typical LD-G/MitoSOX Red dot plots (middle panel). Typical PI/FLICA dot plots (right panel). (B), Treatment with the extract during cryopreservation. Typical LD-G/MitoSOX Red dot plots. LD-G, LIVE/DEAD™Fixable Green Dead Cell Stain; LD-FR, LIVE/DEAD™Fixable Far Red Dead Cell Stain; PI, propidium iodide; FLICA, Fluorescein-Labeled Inhibitor of Caspases.
