# Supplementary material for: Protective Effect of Raphanus sativus Seed Extract on Damage Induced by In Vitro Incubation and Cryopreservation of Human Spermatozoa
Source: Antioxidants (Basel). 2026 Jan 6;15(1):74. doi: 10.3390/antiox15010074 (PMC12837813; doi:10.3390/antiox15010074)
Supplement: Supplementary file 1 [file antioxidants-15-00074-s001.zip › Supplementary Figure S1.pdf]

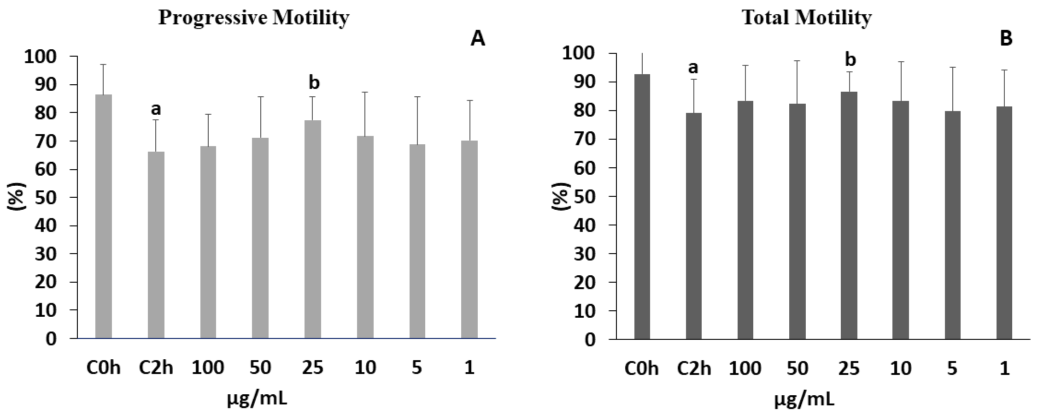

Figure S1: Screening of Raphanus sativus seed extract concentrations (1–100 µg/mL). (**A**), progressive sperm motility: a, *p* (vs. C0h) < 0.001; b, *p* (vs. C2h) < 0.01. (**B**), total sperm motility: a, *p* (vs. C0h) < 0.05; b, *p* (vs. C2h) < 0.05. Data are mean ± SD; Wilcoxon signed-rank test with Bonferroni correction for *p*-value adjustment. C0h, Control before incubation; C2h, control after 2 h of incubation.
