# Supplementary material for: Protective Effect of Raphanus sativus Seed Extract on Damage Induced by In Vitro Incubation and Cryopreservation of Human Spermatozoa
Source: Antioxidants (Basel). 2026 Jan 6;15(1):74. doi: 10.3390/antiox15010074 (PMC12837813; doi:10.3390/antiox15010074)
Supplement: Supplementary file 1 [file antioxidants-15-00074-s001.zip › Supplementary Figure S2.pdf]

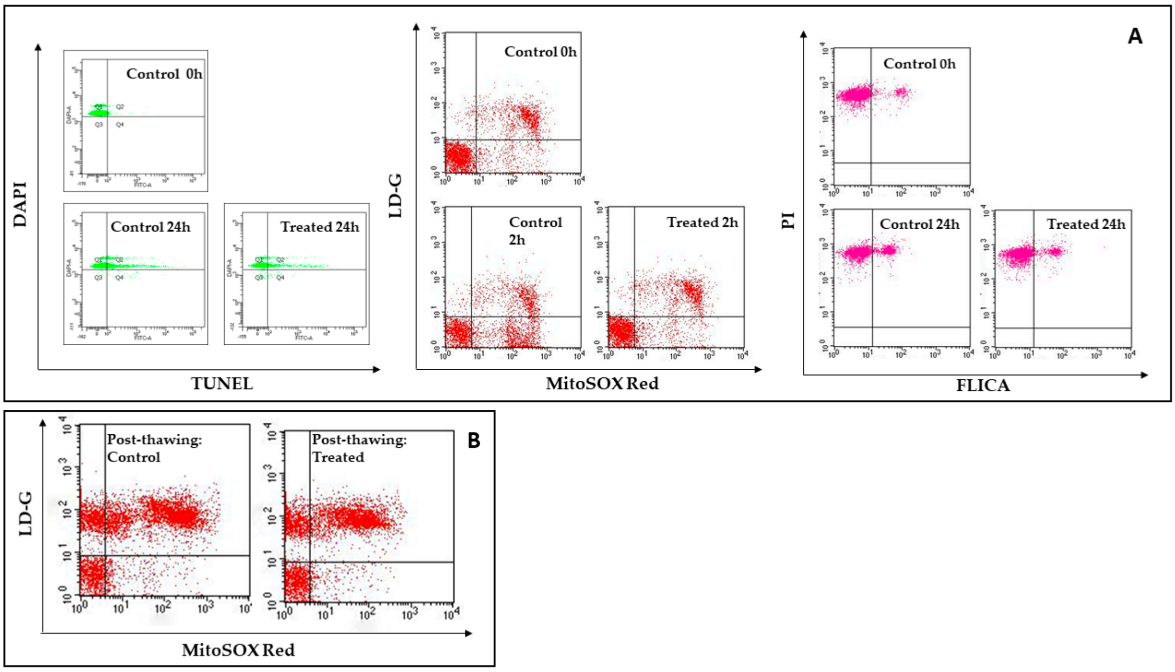

Figure S2. (**A**), Treatment with the extract during in vitro incubation. Typical DAPI/TUNEL dot plots of the viable sperm fraction, obtained after gating of LD-FR negative events (left panel). Typical LD-G/MitoSOX Red dot plots (middle panel). Typical PI/FLICA dot plots (right panel). (**B**), Treatment with the extract during cryopreservation. Typical LD-G/MitoSOX Red dot plots. LD-G, LIVE/DEAD™Fixable Green Dead Cell Stain; LD-FR, LIVE/DEAD™Fixable Far Red Dead Cell Stain; PI, propidium iodide; FLICA, Fluorescein-Labeled Inhibitor of Caspases.
