# Supplementary material for: Protective Effect of Raphanus sativus Seed Extract on Damage Induced by In Vitro Incubation and Cryopreservation of Human Spermatozoa
Source: Antioxidants (Basel). 2026 Jan 6;15(1):74. doi: 10.3390/antiox15010074 (PMC12837813; doi:10.3390/antiox15010074)
Supplement: Supplementary file 1 [file antioxidants-15-00074-s001.zip › Supplementary Table S1.pdf]

Supplementary Table S1. Age, abstinence, and main semen parameters of subjects recruited for experiments of in vitro incubation (n=33)

Age (years)	Abstinence (days)	Volume (mL)	pH	Number (10^6)	Concentration (10^6/mL)	Total Motility (%)	Progressive Motility (%)	Morphology (%)
39.00 [31.00–44.00]	4.00 [3.50–6.00]	4.30 [2.60–4.70]	7.60 [7.60–7.80]	225.75 [141.80–411.95]	66.00 [35.00–103.00]	67.00 [59.00–70.00]	59.00 [51.50–64.00]	4.00 [2.50–7.00]
