# Supplementary material for: Protective Effect of Raphanus sativus Seed Extract on Damage Induced by In Vitro Incubation and Cryopreservation of Human Spermatozoa
Source: Antioxidants (Basel). 2026 Jan 6;15(1):74. doi: 10.3390/antiox15010074 (PMC12837813; doi:10.3390/antiox15010074)
Supplement: Supplementary file 1 [file antioxidants-15-00074-s001.zip › Supplementary Table S2.pdf]

Supplementary Table S2. Age, abstinence, and main semen parameters of subjects recruited for cryopreservation experiments (n=32)

Age (years)	Abstinence (days)	Volume (mL)	pH	Number (10^6)	Concentration (10^6/mL)	Total Motility (%)	Progressive Motility (%)	Morphology (%)
35.00 [32.00–42.00]	4.00 [4.00–6.00]	4.60 [3.70–5.20]	7.80 [7.60–7.80]	313.90 [216.30–414.00]	62.00 [47.00–85.00]	68.00 [62.00–73.00]	62.00 [56.00–66.00]	4.00 [3.00–6.00]
