# Supplementary material for: Protective Effect of Raphanus sativus Seed Extract on Damage Induced by In Vitro Incubation and Cryopreservation of Human Spermatozoa
Source: Antioxidants (Basel). 2026 Jan 6;15(1):74. doi: 10.3390/antiox15010074 (PMC12837813; doi:10.3390/antiox15010074)
Supplement: Supplementary file 1 [file antioxidants-15-00074-s001.zip › Supplementary Table S3.pdf]

Supplementary Table S3. Recovery rate of the indicated parameters by the TYB and SF procedures for cryopreservation

	**TYB**	**SF**	**p***
**Progressive Motility (%)**	0.18 [0.13–0.29]	0.07 [0.03–0.16]	0.001
**Total Motility (%)**	0.44 [0.31–0.56]	0.24 [0.15–0.38]	<0.001
**Viability (%)**	2.00 [1.76–2.60]	0.36 [0.28–0.44]	<0.001
**sDF (%)**	2.00 [1.76–2.59]	1.88 [1.71–2.47]	0.683
**sOS (%)**	2.25 [1.42–4.14]	2.75 [1.59–3.86]	0.049

*Wilcoxon signed-rank test
